# Machining of Fibre Reinforced Plastic Composite Materials

**DOI:** 10.3390/ma11030442

**Published:** 2018-03-18

**Authors:** Alessandra Caggiano

**Affiliations:** 1 Fraunhofer Joint Laboratory of Excellence on Advanced Production Technology (Fh-J_LEAPT UniNaples), 80125 Naples, Italy; alessandra.caggiano@unina.it; Tel.: +39-081-7682371; 2 Department of Industrial Engineering, University of Naples Federico II, 80125 Naples, Italy

**Keywords:** composite material, fibre reinforced plastic (FRP), machining

## Abstract

Fibre reinforced plastic composite materials are difficult to machine because of the anisotropy and inhomogeneity characterizing their microstructure and the abrasiveness of their reinforcement components. During machining, very rapid cutting tool wear development is experienced, and surface integrity damage is often produced in the machined parts. An accurate selection of the proper tool and machining conditions is therefore required, taking into account that the phenomena responsible for material removal in cutting of fibre reinforced plastic composite materials are fundamentally different from those of conventional metals and their alloys. To date, composite materials are increasingly used in several manufacturing sectors, such as the aerospace and automotive industry, and several research efforts have been spent to improve their machining processes. In the present review, the key issues that are concerning the machining of fibre reinforced plastic composite materials are discussed with reference to the main recent research works in the field, while considering both conventional and unconventional machining processes and reporting the more recent research achievements. For the different machining processes, the main results characterizing the recent research works and the trends for process developments are presented.

## 1. Introduction

Composite materials are made of two or more different materials and deliver properties that are not achievable by any single material component. In the composite, one material acts as the matrix and at least one other material has the role of reinforcement. The matrix material deploys diverse functions: reinforcement material protection; stress distribution among reinforcement materials; and, form definition of the manufactured composite part. The reinforcement materials ensure high mechanical properties and perform the strengthening of the composite in pre-selected directions. The composite material properties are determined by the kind of reinforcement and matrix materials, the reinforcement material geometry (short fibres, long fibres, fabric), the percent content of reinforcement, and matrix materials. The matrix material type allows for the classification of composites into three main classes: plastic matrix composites; metal matrix composites; and, ceramic matrix composites. The most extensively utilized composite materials class for industrial applications is represented by plastic matrix composites. The latter are the focus of this article with particular reference to composites made of soft and ductile polymeric matrixes reinforced by high strength/high modulus brittle fibres.

Fibre reinforced plastic (FRP) composites found their initial industrial field of application in the aeronautical industry, but to date they find usage in a very large number of industrial sectors: aerospace, sporting goods, nautical, construction, medical, automotive, train manufacturing, etc.

FRP composites deliver higher strength-to-weight and modulus-to-weight ratios in comparison with metal materials and provide superior resources and opportunities for innovative design. For these valuable reasons, new industrial applications keep proliferating, and, accordingly, new production technologies become increasingly required in order to ensure the effectiveness and economical sustainability of composite materials parts manufacturing.

One of the main issues that are related to FRP parts manufacturing is that they are definitely difficult to cut [1,2]. When an amount of composite material needs to be removed with traditional cutting tools to obtain the final composite, a very low machinability behaviour is evidenced. This shortcoming aspect of FRP parts manufacturing is not yet solved. Intrinsically, FRP composites are nonhomogeneous, anisotropic, and strengthened by highly abrasive reinforcements. These structural characteristics indeed make them difficult to machine: significant material damage is introduced in the part, surface integrity is impaired, part quality and dimensional accuracy are unsatisfactory, high tool wear rate is experienced, and unacceptable scrap rate levels are verified.

The phenomena underlying material removal mechanisms of composite materials are substantially different from those characterizing the machining of metals. Lopresto et al. [3] described the mechanisms of chip formation in cutting of FRP composites laminates and identified a number of parameters, like tool material, tool geometry, depth of cut, and fibre orientation, which play a fundamental role in composite materials machining. The mechanisms of chip formation in FRP composites are based on different failure modes occurring simultaneously and are predominantly governed by the fibre orientation angle, i.e., the angle between the fibre orientation and the cutting direction, rather than tool rake angle, governs the mechanism of chip formation and strongly affects the cut surface quality. For fibre orientations >60°, the cut surface quality was unacceptable. The studies on chip formation mechanisms evidenced two main kinds of induced surface integrity damage: matrix cracking parallel to fibres and out-of-plane displacement of the material to be removed [3].

The fibre orientation with respect to the cutting direction has also a strong influence on the cutting forces, the evolution of which is hard to model due to the complex chip formation.

As regards the cutting tools employed in machining of FRP composite parts, adequate strength, toughness, hardness and thermal shock resistance are needed to withstand the fluctuating mechanical and thermal loads. Two classes of tool materials are generally used: hard materials like cemented carbides, coated carbides and ceramics; superhard materials like cubic boron nitride (CBN), CVD diamond and polycrystalline diamond (PCD) [3].

In the scientific literature, several authors dealt with machining of composite materials, which is still nowadays critical due to their low machinability behavior, and a number of review papers have been presented. In 1997, Komanduri [4] provided a broad overview on the various issues that are involved in machining of fibre reinforced composites, including both conventional and unconventional processes. Since then, a number of new process developments, innovative tool materials and geometries, and advanced machine tools have been established. Teti et al. [1], in 2002, provided a review on machining of composite materials, including plastic matrix composites (PMC), with particular reference to fibre reinforced plastics, metal matrix composites (MMC), and ceramic matrix composites (CMC), basically focusing on traditional machining processes, such as drilling, milling, turning, and orthogonal cutting. Gordon et al. [5] presented a review of research on the cutting of fibre reinforced polymer composites and medium-density fibreboard. Most of the presented research studies focused on traditional metal cutting tools and techniques and concentrated on the chip formation process and cutting force prediction with unidirectional FRP materials. A review of research studies on the prediction of cutting forces for medium-density fibreboard was also presented. Dandekar et al. [6] focused on modeling of machining of composite materials with a focus on turning processes, discussing modeling of both fibre reinforced and particle reinforced composites. Abrao et al. [7] presented a literature survey on the machining of composite materials, more specifically on drilling of glass and carbon fibre reinforced plastics, investigating aspects such as tool materials and geometry, machining parameters and their influence on thrust force and torque, and the quality of the holes. More recently, in 2016, Lopresto et al. [3], presented a review on cutting of fibre reinforced plastic composite materials, focusing on basic studies to understand chip formation mechanisms, machined surface morphology and integrity, cutting forces, as well as tool wear development.

In the present review, following the approach proposed by Komanduri [4], the main issues concerning the machining of fibre reinforced plastic composite materials are discussed with reference to more recent research studies in the field, while considering both conventional and unconventional machining processes and reporting the more recent research achievements. For the different machining processes, the main results characterizing the recent research works and the trends for potential future developments are presented.

## 2. Basic Studies on Orthogonal Cutting

Orthogonal cutting forms the basis of all machining operations: therefore, several research efforts have been spent to understand the material removal mechanisms that are occurring during orthogonal cutting of FRP composites to support the comprehension of the main FRP conventional cutting processes, such as drilling, milling, trimming, etc. [1].

In [8], low speed orthogonal cutting tests at different fibre cutting angles were performed on carbon Fibre reinforced plastic (CFRP) specimens using a conventional HSS tool. While no damage was verified in the range *θ* = 0°–30°, all of the other fibre orientations provided longitudinal and transverse matrix cracking, determining splits deeply penetrating into the workpiece in the side layers. The fibres at the edges of the specimen were bent out-of-plane, passing uncut under the tool, and only the material located around the mid-thickness was effectively removed. Rather than depending on the particular fibre and matrix materials or cutting speed used, this type of failure seemed to be due to the intrinsic anisotropy of unidirectional composites.

In [9], a study on the characterization of subsurface strains during orthogonal cutting of CFRP using a non-contact, full-field strain measurement technique to monitor the laminate in-situ during orthogonal cutting was presented. Chip formation mechanisms and reaction forces during cutting were measured and correlated to the cutting mechanisms for different types of CFRP specimens. Chip formation mechanisms were found to be dependent on the depth of cut and fibre orientation for 0° and 45° specimens. Chips were continuous for 90° and 135° specimens for both the depths of cut. Cutting forces were found to be dependent on fibre orientation *θ*, but independent from cutting speed. Increasing the depth of cut resulted in increased cutting forces. Strains in the subsurface region on the flank side showed a typical behavior with a compressive *ε*_xx_ in front of the cutting tool and tensile *ε*_xx_ behind the cutting tool.

In [10], orthogonal cutting experiments were carried out on unidirectional CFRP with purposely designed orthogonal cutting tools at a cutting speed of 0.5 m/min. A high-speed camera was employed to investigate the chip formation mechanisms and chip morphology. Two main factors were found to influence the chip morphology, i.e., the fibre orientation angle (*θ*) and, secondly, the depth of cut (DOC). The chips were powder-like or ribbon-like, under small DOC (≤0.1 mm), but became blocky with higher DOC. In the case of 15° ≤ *θ* < 75°, chips were produced by the matrix-fibre interface shearing along the fibre direction, while for *θ* > 75°, the chip was produced due to bending fracture, leading to fibre bouncing-back that degrades the surface roughness of machined surface.

Qi et al. [11] developed a theoretical model for predicting the cutting and thrust forces in the orthogonal cutting of UD-CFRP with a fibre orientation angle 0° ≤ *θ* ≤ γ_α_ + 90°, where γ_α_ is the rake angle of the orthogonal cutting tool. The deflection of a representative volume element, composed of a single fibre and the surrounding matrix, was analyzed while considering the effect of the surrounding materials based on the minimum potential energy principle. The critical force in the cutting edge that causes fracture of the representative volume element was obtained. The actual force is a complex combination of various component forces according to different mechanisms: by taking into consideration slipping, peeling, and bounding mechanisms in three different deformation areas, a force prediction model of UD-CFRP orthogonal cutting was established for the specified fibre orientation angles.

Chen et al. [12] constructed a theoretical model to predict the cutting and thrust forces in orthogonal cutting of UD-CFRP under the entire range of fibre orientation varying from 0° to 180° based on the theory of beams on elastic foundation and the principle of minimum potential energy. The specific relationship between cutting parameters, such as fibre orientation, depth of cut, rake angle, and cutting and thrust forces, were accurately expressed in the model. It was found that fibre orientation has the most significant influence on cutting and thrust forces, followed by depth of cut, and finally, rake angle. The model can be used for process parameters optimization, tool performance improvement, and cutting cost reduction, and can be extended to FRP machining processes, such as drilling, milling, and trimming.

In [13], tool condition monitoring was carried out through acquisition and analysis of acoustic emission (AE) that was generated during orthogonal cutting using HSS tools on different types of composite materials: UD glass Fibre reinforced plastic (GFRP), UD carbon Fibre reinforced plastic (CFRP), and sheet molding compound (SMC). Decision making on tool wear state was performed via supervised neural network data processing of AE spectrum features for pattern recognition in multi-dimensional feature spaces. Different results were obtained, according to the composite type: tool wear discrimination was reliably achieved for GFRP, but not as robustly for CFRP and SMC [14].

All of the presented research studies on orthogonal cutting of fibre reinforced composite materials converge to the conclusion that different cutting mechanisms occur when machining such anisotropic and inhomogeneous materials. Moreover, the studies agree on the identification of the main parameter governing the mechanisms of chip formation and the cutting and thrust forces, which is the fibre orientation angle, i.e., the angle that is formed between the fibres and the cutting direction. Unfavorable fibre orientation angles lead to unacceptable machined surface quality, with mechanisms such as fibre bending fractures and the development of cracks into the composite.

## 3. Conventional Machining Processes

### 3.1. Turning

Turning, along with drilling, milling, and trimming, is one of the most widely employed cutting processes for the machining of FRP composite materials and is applied to rotation-symmetric parts, such as shafts, tubes, gears, spindles, etc. [1,15].

Many research efforts have been spent to investigate the effects of cutting parameters as well as cutting tools geometry and materials with the aim to determine suitable cutting conditions for effectively applying turning processes to the various FRP composite materials.

Davim and Mata [16] studied the machinability of two different glass fibre reinforced plastic composite materials, one manufactured by filament winding and the other by hand lay-up, in turning processes using polycrystalline diamond (PCD) cutting tools. A statistical procedure, based on orthogonal arrays and analysis of variance, was applied to study the effect of different cutting parameters on surface roughness and specific cutting pressure. As regards the cutting parameters, feed rate proved to have the highest influence on surface roughness (*R_a_*) and specific cutting pressure (*K*s). Moreover, the hand lay-up process provided lower specific cutting pressure values, and smaller surface roughness when compared to filament winding, yielding to the best machinability index under the optimal cutting parameters (*v*_c_ = 400 m/min, *f* = 0.1 mm/rev).

In [17], the authors presented a study on the optimization of surface roughness in turning of GFRP tubes with PCD cutting tools, using multiple analysis regression (MRA) with the aim to establish the optimal cutting parameters to achieve specified surface roughness values (*R_a_* and *R_t_*/*R*_max_). It was shown that surface roughness increases with feed rate and decreases with cutting speed.

In [18], the turning process of unidirectional (UD) GFRP using cermet tools was investigated through an experimental campaign in which cutting speed (Vc), feedrate (f), and depth of cut (a), were varied according to a three-level full factorial experimental design technique, whereas the cutting direction was held parallel to the fibre orientation. An artificial neural network (ANN) and response surface model were developed to predict surface roughness, providing good agreement between the predictive models results and the experimental measurements, with maximum test errors of about 6%.

In [19], turning of UD-GFRP rods obtained by pultrusion using cermet tools was studied to correlate the surface roughness to a number of machining parameters including cutting speed, feed rate, depth of cut, and tool geometry. The cutting conditions were varied, as follows: cutting speeds = 75, 100, 125 m/min; rake angle = 6° and 18°; tool radius = 0.4 and 0.8 mm; depth of cut = 0.6, 0.9, 1.2 mm; feed rate = 0.2, 0.3, 0.4 mm/rev; and, relief angle = 7°. The surface roughness was found to decrease with increasing cutting speed as well as with an increase of the tool radius while it increased with an increase of the feed rate and of the rake angle. Contrarywise, the cutting depth did not significantly affect the surface roughness.

In [20], a new multicriteria optimization approach to minimize cutting forces and maximize the material removal rate was proposed for the selection of the optimal machining parameters in turning of UD-GFRP rods produced by pultrusion with polyester and E-glass (82.27% glass content). When compared to the results obtained by the previous study, which considered only surface roughness as output, higher feed values were selected by the optimization approach, since although slow feed values provide better surface roughness, higher feed values provide better results when the material removal rate is also considered.

Palanikumar et al. [21] presented a study on the influence of cutting parameters on surface roughness parameters in turning of GFRP tubes (polyester matrix with 65% of glass fibre content) with PCD tools (rake angle 6°, clearance angle 11°, edge major tool cutting 91°, and cutting edge inclination angle 0°). Empirical models were developed to correlate the surface roughness parameters with feed and cutting speed. The analysis of surface roughness parameters with respect to experiments was carried out using area graph, and the analysis of cutting parameters was carried out using three-dimensional surface plots. The results obtained were consistent with the other studies in the literature, confirming that surface roughness increases with the increase of feed rate and decreases with the increase of cutting speed.

In [22], an attempt was made to assess the main factors affecting tool wear in the turning of GFRP composites through a procedure integrating response table and effect graph, normal probability plot, interaction graphs, and analysis of variance (ANOVA). The factors that were considered were cutting speed, fibre orientation angle, depth of cut, and feed rate. The results showed that the factor that mainly affects the tool flank wear is the cutting speed, followed by feed rate. Moreover, the interaction between cutting speed and depth of cut was found to have a major influence, when compared with other interactions, on tool flank wear in the turning of GFRP composites. The optimization procedure also allowed for predicting the tool flank wear in GFRP turning within the range of parameters considered in the study.

In [23], the flank wear in the turning of CFRP composite pipes was analysed for different tool materials: uncoated sintered carbide, diamond-coated silicon nitride, and PCD tools with 5 μm grain size and cobalt binder. After turning the same length with a cutting speed 100 m/min, feed rate 0.1 mm/rev, and depth of cut 0.5 mm, the flank wear of the uncoated sintered carbide tool is notably higher than the one for the PCD (4.5 times) and the diamond coated silicon nitride tools (3.5 times), thus confirming the lower performance of sintered carbide tools in comparison with the high performing diamond-based tools [24].

The research studies carried out on turning of fibre reinforced plastic materials highlighted the need to employ hard material tools, such as cermet tools, in the case of GFRP composites, and superhard materials, such as PCD tools, for both GFRP and CFRP composites. These tool materials are characterized by adequate strength, toughness, hardness, and thermal shock resistance. As regards the cutting parameters, it is worth mentioning that the results obtained by the different research studies agree on one fundamental aspect: when considering surface roughness as the main output quality parameter to be optimized, slow feed values must be selected for the turning process. 

### 3.2. Milling

Milling processes on FRP composite parts are generally performed as a corrective end-machining operation distinguished by a low ratio of material removed to total part volume compared to milling of metal parts. The selection of the proper tool and machining parameters is governed by several factors, including fibre type, reinforcement architecture, and matrix volume fraction [1]. Sintered carbides, CBN, and PCD cutting tool materials are the most widespread tool materials for FRP milling, as they have high hardness and high thermal conductivity.

A study on tool life performance of uncoated and diamond coated carbide end mills in CFRP milling was reported in [24], showing that for diamond coated tools, higher feed rate and smaller coating thickness cause tool wear through fracture and delamination of the diamond coating. The flank wear of the diamond coated tools was significantly lower than the one of the uncoated tool and tended to increase at higher feed rate. The lower feed rate generated a smaller chip per tooth, and, consequently, lower cutting forces and less severe impact on the cutting edge, thus enhancing the diamond coating resistance by reducing its chipping and delamination. Tool life was also longer for increased diamond coating thickness (10 μm vs. 20 μm), also at low feed rate. The thicker coating performed poorly at high feed rate, probably due to higher internal stresses. The role played by the feed rate in controlling the performance of diamond coated tools during FRP milling was highlighted: higher feed rate and smaller coating thickness were found to cause tool wear by fracture and delamination of the diamond coating.

The employment of DLC (Diamond-Like Carbon)-coated carbide end mills with different helix angles for milling of CFRP parts was investigated in [25]. The flank wear of the end mill shows that tool wear is strongly dependent on the fibre orientation angle *θ*. The flank wear of the high helix end mill (β = 60°) was 50% smaller than the one of the standard end mill (β = 30°) for all of the fibre orientation angles. A relatively large flank wear was obtained for *θ* = 90° and *θ* = −45°. For these orientation angles, the fibres bend and fracture during cutting so that the friction between flank face and fibres increases due to the fibre spring back. To reduce tool wear and improve machined surface integrity, inclination milling with high helix angle end mills, in which the resultant cutting force is parallel to the CFRP surface, was proposed.

Sheikh-Ahmad et al. [26] utilized a mechanistic modeling approach to predict cutting forces and simulate the milling process of unidirectional and multidirectional FRP composites using a straight cutting edge. Specific energy functions were developed by multiple regression analysis and committee neural network approximation of milling force data and a cutting model was developed based on the energy functions and the cutting geometry. Both of the models were capable of predicting the cutting forces in milling of unidirectional and multidirectional composites over the entire range of fibre orientations from 0 to 180°. The neural network model provided the best prediction accuracy and was able to smooth the noisy data and capture the inherent non-linearity in the experimental data.

Gara and Tsoumarev [27] studied the slotting of CFRP composites using knurled tools and defined a model of the transverse and longitudinal arithmetic average surface roughness. They observed that: feed per tooth has the highest influence on surface roughness; transverse roughness does not depend on cutting conditions, but only on tool geometry; longitudinal roughness depends on tool geometry and cutting conditions; and, knurled tool fine toothing is the suitable tool for the slotting of CFRP material, as it generates less damage to the laminate plate.

In [28], an experimental study of slotting of multidirectional CFRP laminate using three micro grain carbide burr tools with different geometries and an infrared thermograph camera was carried out to investigate tool-workpiece contact point temperature, chip temperature, machined surface damage, subsurface defects, and tool degradation. Empirical models were established to show the dependence of cutting temperature on tool geometry and cutting conditions. Cutting speed was found to be the most influencing factor with respect to the cutting temperature (due to the growth of the friction between cutting tool and machined surface), followed by feed per tooth. With the increase in cutting speed, the chip temperature rises. When the feed per tooth is increased, the temperature also rises. It was also observed that the heat generated during slotting is removed mainly by chips, and the chip temperature is greater than the tool-workpiece contact temperature of about 18.5 °C on average.

Azmi et al. [29] studied end milling of GFRP composites to assess their machinability taking into consideration surface roughness, tool life and machining forces. Taguchi analysis, combined with statistical analysis of variance (ANOVA), was performed to quantify the effects of spindle speed, feed rate and depth of cut on the machinability indices. Multiple regression analysis (MRA) was employed to establish parametric relationships between the experimental parameters and the machinability parameters. Both results showed that feed rate is the governing factor affecting all the machinability parameters. Feed rate has the most dominant role in influencing the surface roughness, followed by spindle speed. The dominant effect of feed rate may be attributed to the different mechanisms of chip formation at various feed rates. The tool life of the end mill cutter was mainly influenced by the feed rate and spindle speed. The influence of depth of cut on surface roughness and tool wear was negligible. The machining force was notably affected by feed rate and depth of cut.

In [30], a finite element model was used to investigate the cutting forces, chip formation mechanism, and machining damage during flat end milling of unidirectional CFRP. The developed two-dimensional (2D) finite element model, validated with experimentally measured forces and SEM images of the machined surface, highlighted the role of the fibre orientation angle, i.e., the angle between the carbon fibres and feed direction. The cutting forces showed different profiles for diverse fibre cutting angles. At low tool rotation angles (30°–60°), the fibre compressive failure and matrix crushing progressed in the fibre direction until complete chip formation, while at higher tool rotation angles (≥90°), the chip was formed in matrix crushing mode. The extension of machining damage significantly depends on the fibre orientation: at 0° fibre orientation angle, the compressive damage extended in the direction of fibres; for 45° and 60° tool rotations, this failure mode affected a large zone of uncut material. The matrix cracking failure also affected a large zone of uncut material below the tool for all tool rotation angles.

In [31], slot milling of CFRP composites was investigated with the aim to develop a mechanistic milling force model for both unidirectional and multi-directional CFRP laminates while considering the instantaneous chip thickness, fibre orientation angle, and cutting speed. In a cutting cycle, the tangential and radial forces vary with the combined effects of instantaneous fibre orientation angle and chip thickness. The 0°/180° fibre orientation angle displayed the largest radial force and specific cutting energy, and 135° the largest tangential forces and specific cutting energy. At 45° fibre orientation angle, the smallest tangential and radial forces as well as specific energies were observed. The 90° fibre orientation angle was identified as the critical angle above which severe fluffing/delamination on top layer is induced due to fibre bending, and below which little or no fluffing is induced due to fibre crushing. The damage proved to increase with increasing speed for a fixed feed per tooth.

Uhlmann et al. [32] studied the use of high cutting speeds in the milling of CFRP, showing that a fundamental change in chip formation mechanisms occurs. Higher cutting speeds lead to a decrease in process forces that can be employed to prolong tool life or increase the feed rate at the same tool life. A study on milling of CFRP composites at very high cutting speed (up to 200 m/min) was also carried out in [33] for specific fibre orientation angles, showing the difficulty in achieving a satisfactory cut quality.

The influence of Minimum Quantity Lubrication (MQL) parameters on machining performance in milling of CFRP laminates was studied in [34]. Flank wear was reduced by 30% when compared to pressurized air and 22% as compared to dry and flood coolant. High air flow rate and low oil flow rate provided the longest tool life and the lowest machining error.

Hintze and Hartmann [35] investigated material integrity of FRP composites in the milling process. Delamination and fibre protrusions are the typical damages that can be generated by the milling process, and both occur at different ranges of the fibre orientation angle. Top layer delamination results predominantly from the bending of fibre bundles either within the laminate plane or perpendicular to it, depending on the fibre orientation angle. Fibre protrusions are always associated with top layer delamination. To improve the edge quality by preventing the propagation of delamination in the top ply, a procedure that is based on initial scoring along the contour before cutting, performed by grinding or laser cutting, was proposed in [36,37].

Szallies et al. [38] proposed low frequency (<30 Hz) oscillated milling of unidirectional CFRP as an alternative to conventional milling to improve surface quality. Using a special solid carbide tool, the produced delamination was smaller than in the case of conventional milling, regardless of the fibre orientation angle.

The presented research studies recognize that typical damages, such as delamination and fibre protrusions, can be generated by the milling process at different ranges of fibre orientation angle. To reduce these defects, different solutions have been proposed. Some authors suggested to perform an initial scoring along the contour via grinding or laser cutting. Others proposed oscillated milling as a new process development to reduce delamination, as the very recent research studies reported a significant improvement of the milled surface quality.

By summarising the main trends regarding the milling of FRP composite materials, the investigation of high speed milling is a promising field of research, as the recent experimental results showed that higher cutting speeds lead to decreased process forces, allowing for prolonged tool life or increased feed rate. Another interesting opportunity is the employment of minimum quantity lubrication which may allow to prolong tool life and to reduce the environmental impact related to a more intensive use of cutting fluids.

### 3.3. Drilling

Drilling process applied to FRP composites has been studied by numerous researchers as it is one of the most widely employed machining processes of FRP materials due to the extensive utilization of mechanical joints, such as rivets, instead of welded or bonded joints. The main challenges in FRP drilling are due to rapid tool wear and damage to material integrity and surface quality.

Research and review studies in drilling of composite materials were reported in [39], in particular with reference to the influence of machining parameters and tool geometry on the delamination. An adequate selection of drilling tools and machining parameters to extend the life cycle of the FRP laminates as a consequence of enhanced reliability [40].

Several types of tools, characterised by different geometry and material, have been employed to drill FRP composites. A comprehensive analysis of delamination in using various drills, including traditional twist drills and special drills as candle stick drill, saw drill, core drill, and step drill was illustrated in [39]. In [41], high performance sintered carbide drills for CFRP composite drilling were examined. TiN and DLC coatings were employed to reduce the high wear rate of the sintered carbide drills, and the coating performance was studied in terms of material damage and thrust force and torque that was generated during processing. The damage produced by drilling was due to spalling, chip-out, and matrix cracking. The coatings were not found to reduce either tool wear or damage to the composite.

In [42], the wear of uncoated and diamond-coated carbide tools in CFRP composite drilling was revealed to be correlated with the axial force that was applied to the cutting edge multiplied by the length of the contact between cutting edge and work material. For uncoated carbide tools, this correlation is a power law function, whereas for diamond-coated carbide tools, the correlation is linear at the beginning and becomes a power law function at the end. A phenomenological model of axial load for tool wear prediction was proposed, allowing for forecasting the simultaneous development of axial load and tool wear. The diamond coating on the carbide drill was shown to be extremely advantageous, yielding a tool life 10 to 12 times higher than the uncoated carbide drill for cutting speeds three times higher (170 m/min versus 56 m/min) [3].

Tsao and Hocheng [43] developed an analytical approach based on linear elastic fracture mechanics to identify the process window of chisel edge length relative to drill diameter for delamination-free drilling. The experimental results indicated that the critical thrust force is reduced with the pre-drilled hole, while the drilling thrust is largely reduced by cancelling the chisel edge effect. By controlling the ratio of chisel edge length, it is possible to drill medium to large holes in composite laminates at a higher feed rate without delamination damage.

In [44], machinability maps were introduced to select the optimal drilling conditions satisfying the requirements of surface integrity, surface roughness, hole circularity, and hole diameter error, during drilling with 5 mm diameter drills, 1500–15,000 rpm rotational speed, and 0.02–0.8 mm/rev feed rate. The thrust force trend and the correspondent delamination damage were in agreement with the wear zones and it was observed that, beyond the primary wear zone, undersized holes were generated as a consequence of increasing tool flank wear.

**Figure 1:**
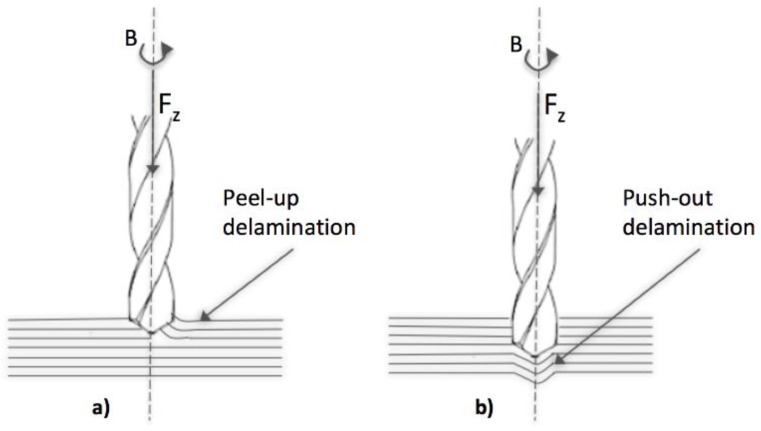
(**a**) Peel-up and (**b**) push-out delamination induced by drilling of fibre reinforced plastic (FRP) laminates.

The authors in [45] investigated high speed drilling in CFRP thin laminates using K20 carbide drill by varying the drilling parameters, such as spindle speed and feed rate, to determine the optimum cutting conditions. The hole quality parameters, including hole diameter, circularity, peel-up delamination, and push-out delamination (Figure 1), were analyzed. It was shown that feed rate has a greater influence on thrust force, push-out delamination, and diameter of the hole (lower feed rates reduce thrust force and push-out delamination, higher feed rates result in holes closer to the nominal diameter), while spindle speed is one of the major determinants of the circularity of the drilled hole. Neither spindle speed nor feed rate had any visible influence on peel-up delamination within the tested range. The peel-up delamination was lesser when compared to push-out delamination throughout the study of tool life.

Tsao and Hocheng [46] presented a study on the use of back-up plates employed to support and counteract the deflection of the composite laminate leading to exit side delaminations in drilling. The aim of their models was to predict the effect of the back-up plate on delamination in drilling CFRP using a saw drill and a core drill. Based on the proposed models, both the saw drill and the core drill with backup offer a higher critical thrust force than those without backup, thus allowing for operating at a larger feed rate without delamination damage.

Phapale et al. [47] presented a comprehensive study on the cutting mechanism and the relative effect of cutting parameters on delamination during CFRP drilling. Thrust force and torque data are acquired for analyzing the cutting mechanism, initiation and propagation of delamination, and the identification of critical thrust force below which no damage occurs. It was observed that the thrust force is strongly dependent on the feed rate, probably due to higher undeformed chip thickness at higher feed rates. The delamination is a function of both feed and spindle speed. The effect of feed is amplified at higher rotational speeds.

With the aim to support on-line decision making on tool change execution through cognitive tool wear prediction and hole quality assessment, Caggiano et al. [48] implemented a multiple sensor process monitoring procedure in drilling of CFRP/CFRP stacks for the assembly of aircraft fuselage panels. Thrust force, torque, and acoustic emission RMS signals were acquired during experimental drilling tests with 6.35 mm diameter carbide drills with different rotational speed (2700, 6000, and 9000 rpm) and feed conditions (0.11, 0.15, 0.20 mm/rev). A correspondence between hole diameter error and exit delamination factor with tool wear level was observed: in particular, a tool wear threshold, VB = 0.04 mm, was identified, in proximity of which unacceptable hole quality is generated. This value can be used as a threshold to determine the need for tool change via cognitive on-line prediction of tool wear during drilling.

Davim et al. [49] presented a novel technique using digital analysis to measure the adjusted delamination factor (Fda), and showed that taking into account the damage area in the delamination factor allows for a better characterization of delamination after drilling composite materials.

Abrão et al. [50] investigated the influence of the cutting tool geometry and material on the thrust force and delamination produced when drilling a glass fibre reinforced composite, using four drill bits with different geometries and materials. Lower thrust force was recorded using a carbide drill with two cutting edges, while the highest thrust force was recorded with carbide drill with three cutting edges and a point angle of 150°, which was shown to be detrimental. Thrust force was elevated as feed rate was increased due to the elevation in the shear area, while the influence of cutting speed on thrust force was negligible.

Gaitonde et al. [51] studied the effects of process parameters on delamination during high-speed drilling of CFRP composite, revealing that the delamination tendency decrease with increase in cutting speed. The analysis of the experimental results showed that high-speed cutting plays a major role in reducing damage at the entrance of hole, and the combination of low feed rate and point angle is also essential in minimizing delamination during the drilling of CFRP composites.

Campos Rubio et al. [52] employed high speed to realize high performance drilling of glass fibre reinforced plastics (GFRP) with reduced damage. Also in this case, delamination decreased with elevated spindle speed, probably due to the softening of the matrix. To obtain larger material removal rates and minimal delamination, higher spindle speeds should be used when drilling GFRP.

Krishnaraj et al. [53] focused on the optimal drill point geometry for minimizing the drilling forces and the subsequent damage, and investigated the effects of drill points in drilling at high spindle speed with different drill geometries, like standard twist drill and multifacet drill.

The emerging process of orbital drilling (OD) can contribute to reduce or eliminate the delamination and thermal damage defects that are produced when drilling of composites.

**Figure 2:**
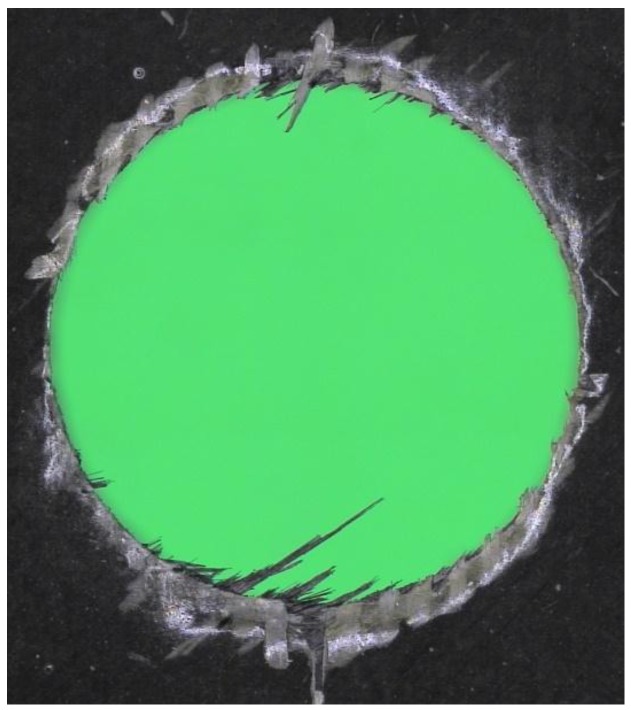
Carbon Fibre reinforced plastic (CFRP) drilled hole exit damage: delamination, spalling and uncut fibres.

A comparison between conventional and orbital drilling of heavy-to-cut unidirectional CFRP parts with diamond coated tools was presented in [54], focusing on workpiece damages, tool wear, bore diameter variances, as well as cycle times. Orbital drilling generated a better hole quality with lower process forces, but required a more complex/dynamic machine tool and longer process times. Up to three times higher axial feed forces occur in conventional drilling, when compared to orbital drilling where the axial feed forces, after initial wear, remain constant. Significantly less hole exit damages (spalling, delamination, or uncut fibres, see Figure 2) and less bore channel damages (fibre cracks, pull-out, and bending) are generated in orbital drilling, but the process time is twice the process time of conventional drilling.

Drilling is probably the machining process that received most attention by researchers, due to its wide employment in industry for the realization of mechanical joints. All of the research studies emphasize that critical damages, such as peel-up and push-out delamination, are generated by drilling due to high thrust forces. Several research studies were focused on the selection of the proper process parameters, as well as tool geometry and material with the aim to reduce such damages. It was recognized by several authors that feed rate has a greater influence on thrust force, push-out delamination, and diameter of the hole. Other research studies focused on the suitable methodologies to measure these defects and properly characterize the hole quality. High speed drilling was investigated, showing promising results in terms of delamination reduction. To reduce or eliminate the delamination and thermal damage defects that are produced when drilling composites, recent trends in drilling of FRP composites are characterised by the emergence of orbital drilling processes, requiring however longer processing times.

### 3.4. Grinding

In [55], the grinding of chopped strand mat (CSM) GFRP laminates was investigated to evaluate the effects of abrasive types, alumina (Al_2_O_3_), and cubic boron nitride (CBN), on the grinding force ratio and surface roughness under various cutting parameters, such as speed, feed, and depth of cut. For both abrasive types, the maximum grinding force ratio was found at low speed, high feed, and low depth of cut. The experimental results indicated that grinding with CBN wheel produced higher grinding force ratios than with the alumina wheel in most of the grinding conditions, therefore being more efficient. The grinding force ratios generally increased with the increasing feed at low speed and depth of cut. The alumina wheel produced smoother surface when grinding at low speed, low feed, and high depth of cut. The CBN wheel, on the other hand, gave smoother surface at high feed and low depth of cut conditions, regardless of speed. Overall, the CBN grinding wheel showed better performances in reducing ground surface roughness than the alumina wheel.

In [56], the grindability of the multidirectional CFRP composites with a layup of [(0°/90°/45°/−45°)3]s was investigated, focusing on chip formation, material removal mechanism, ground surface features, and grinding force characteristics. It was found that the grinding forces for the multidirectional CFRP composites increased nearly linearly with raising the grinding depth and are generally larger than those for the unidirectional CFRP composites under the same grinding conditions. The longitudinal surface roughness of ground multidirectional specimens varied strongly with the measuring location: in correspondence of the 0°, 45°, and 90° plies it was very close to that of the unidirectional ones with the same fibre orientations. Several of chips forms were generated, i.e., a mixture of fine powder, broken fibres, and pieces of broken composite bulks.

Hu and Zhang [57] investigated the grinding performance of epoxy matrix composites reinforced by unidirectional carbon fibres using an alumina grinding wheel. The aim was to understand the effect of fibre orientations and grinding depths on the grinding force and surface integrity. It was found that chip formation, grinding forces, and surface integrity in grinding of FRP with unidirectional fibres are highly dependent on fibre orientations. The fibre orientation of 90° provided the lowest surface roughness, and the grinding depth had minor effect on the surface roughness. A fibre orientation between 120° and 180° proved to be unfavorable, leading to a saw-toothed surface morphology and deep subsurface damage. The depth of the damage-affected zone increased with the increment of grinding depth for all of the fibre orientations studied.

In order to achieve the high-performance machining in grinding of CFRP, the use of an internal coolant through the grinding wheel was proposed in [58]. A vitrified aluminum oxide grinding wheel cup-type grinding wheel was used for face grinding of CFRP. Three different coolant supply systems were tested: dry grinding, coolant supply with external nozzle, and internal coolant through the grinding wheel. The results showed that matrix resin loading on grinding wheel was significantly reduced by the internal coolant supply. The grains of the grinding wheel were able to cut the fibres sharply, without delamination or burr formation on the ground surface, and the surface roughness was reduced. The internal coolant markedly reduced the grinding temperature, keeping it lower than the glass-transition temperature of the matrix epoxy resin and removed the chips from the grinding wheel pores.

In [59], an approach for using diamond grinding tools to machine holes through a drill-grinding process in epoxy carbon laminates was presented. A process simulation was applied to improve the tool layout and to avoid material clogging at the grinding layer. The geometric cutting conditions at the single diamond grains proved to be the most important factors that were influencing both the workpiece load and the process result. Thermographical measurements showed a higher influence of the feed rate on the surface temperatures: in the case of dry machining, the feed rate has to be decreased to avoid a thermal degradation of the resin. To decrease the resulting surface roughness, combination tools with an additional finishing layer on the convex surface were proposed. The investigations on the achievable total drilling length of these diamond tools show the high competitiveness of the drill grinding process when compared to conventional drilling tools.

The use of diamond grinding tools for the drilling of FRP composites represents an interesting alternative to conventional drilling tools. As regards to more conventional grinding operations, although many research studies employed alumina wheels in grinding of fibre reinforced plastic composite materials, it was shown that CBN grinding wheel provide better performances in terms of ground surface roughness, especially when using high feed values.

### 3.5. Other Conventional Machining Processes

Baskaran et al. [60] proposed a fine blanking process as an alternative to conventional drilling for hole making in GFRP laminates of four different reinforcement lay-up sequences: unidirectional [0/0]n, angle ply [0 ± 45]ns, quasi-isotropic [0/45/90]ns, and cross-ply [0/90]n. The observation included tensile and flexural bending strengths of the specimens without hole and with hole by conventional drilling and fine blanking. From the tensile study, it was observed that by inserting a hole at center by drilling, the strength was reduced to one third, and by inserting a hole at the center by fine blanking, the strength was increased nearly 20% than that of drilling.

Wang et al. [61] studied the mechanism that leads to burr formation in edge trimming of CFRP laminates and investigated the influence of fibre cutting angle and cutting edge radius on burr formation. It was found that the fibre cutting angle, rather than the fibre orientation angle, is a key factor to determine burr formation in edge trimming of CFRP laminates, and that the relationship between burr formation and fibre cutting angle greatly depends on the cutting edge radius of the tool. The long burrs tended to be formed when the subsequent fibre cutting angle during the further tool feed motion was less than 90°. Thus, small radial cutting depth and cutting edge radius of the tool are recommended to minimize the burrs.

**Figure 3:**
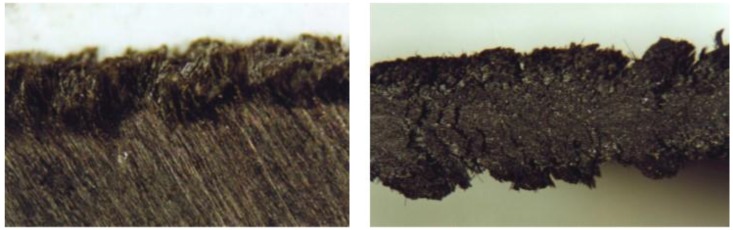
Damaged CFRP pultruded specimen trimmed with the V-shaped tool. Fibre orientation angle *θ* = 120° [62].

To enhance the cut quality in the trimming of unidirectional CFRP composites, Caggiano et al. [62] developed a geometrically improved V-shaped tool and performed experimental tests at a relatively high speed and fixed depth of cut by varying the fibre orientation angle *θ* with respect to the cutting direction in the range 0°–180°. Three parameters were carefully chosen to assess the cut surface: depth of the side splits, D, depth of the internal splits, D_i_, and cut surface roughness, *R_a_*. All of the parameters showed a notable improvement when using the V-shaped tool. To test the effectiveness of a trimming tool, the fibre orientation angle *θ* = 120° was suggested by the authors, since for this fibre orientation angle the highest cutting forces and the poorest cut quality were observed, regardless of the tool geometry (Figure 3).

As an alternative of standard end milling, high speed edge routing of CFRP laminates using single layer electroplated diamond and CBN grinding points was presented in [63]. Greater levels of tool wear, cutting force, and workpiece surface roughness were experienced when employing CBN when compared to diamond abrasives in edge routing of CFRP laminates. In both cases, reduced workpiece damage was observed, but the tool was subject to severe loading by the melted polymer matrix when employing 76 µm abrasive grits under roughing conditions.

## 4. Unconventional Machining Processes

In the last years, several research efforts have been spent to investigate the applicability of unconventional machining technologies, such laser beam processes, electrical discharge machining, or abrasive waterjet machining, due to the typical problems occurring when cutting composites related to high tool wear or material damage due to high mechanical forces.

### 4.1. Laser Beam Machining

Laser beam machining (LBM) represents a promising alternative to the traditional in CFRP cutting technologies, avoiding the mechanical action that may damage the workpiece and the rapid tool wear due to the abrasive fibres. However, the thermal interaction on which LBM is based may result in thermal damages of both matrix and fibres, generating a heat affected zone (HAZ) with an extension that is dependent on the laser source and the process parameters, causing matrix recession, fibres distortion, and delamination.

To reduce the HAZ extent, Hocheng and Pan [64] investigated laser grooving of fibre reinforced composite materials in a cryogenic environment. The anisotropic HAZ associated with cryogenic parameters was experimentally and analytically investigated for both principal-axis and non-principal axis grooving of CFRP laminates. The results showed that the application of cryogenic surroundings could reduce thermal damage in laser grooving by lowering the environmental temperature.

More recently, El-Taweel et al. [65] studied the cutting performance of a CO_2_ laser on Kevlar-49 composite materials. Taguchi analysis was used to identify the effect of laser control parameters, i.e., laser power, cutting speed, material thickness, assistance gas pressure, and laser mode, on the cut quality parameters, i.e., kerf width, dross height, and slope of the cut. The significant parameters and the optimal combination levels of cutting parameters were determined via analysis of variance (ANOVA) and signal-to-noise (S/N) ratio response tables. The results showed that laser power is the most significant parameter affecting the quality of cut parameters. Using the optimum process parameter ranges identified, the Kevlar-49 composites were satisfactorily cut by the CO_2_ laser.

In [66], the layer-by-layer removal of damaged composite material for repair purpose through LBM was proposed, allowing for a significant time-reduction when compared to the traditional manual grinding process. A tripled frequency diode pumped solid state (DPSS) lasers and deflection speeds up to 2 m/s were employed, and the experimental results showed that the HAZ was suppressed and the detachment of fibres from the matrix was avoided.

Zaeh et al. [67] investigated the employment of remote laser cutting for contouring structural CFRP. A positive impact of the HAZ on the mechanical properties was found. Based on FEM simulations, laser contouring with a HAZ = 600 mm almost eliminated the risk of fibre fracture and improved the minimum total fatigue life.

Leone and Genna [68] investigated the laser cutting of 1 mm thick CFRP plates by means of a 150 W Nd:YAG pulsed laser to study the influence of process parameterson, the kerf geometry, and the HAZ. The experimental results showed that the Nd:YAG laser was able to cut the CFRP plate with speed up to 12 mm/s using an average power of about 95 W. An accurate selection of the process parameters is necessary to obtain the maximum cutting speed and a narrow HAZ. A relation between the HAZ extension and the process parameters was determined, showing that the HAZ extension is related to the spot overlap through an exponential law which depends on the combination of pulse energy and frequency. For a fixed average power, high pulse energy and low overlapping factor produce lower HAZ.

A potential option for improving laser cut quality of composites was proposed in [69] and consisted in the introduction of absorbent soot particles into the resin matrix, allowing for with a four-fold decrease in workpiece defects reported based on preliminary experimental trials.

All of the research studies agree that the main challenge in laser beam machining of fibre reinforced plastic composites is related to the generation of a heat affected zone, causing matrix recession, fibres distortion, and delamination. An accurate selection of the process parameters, with particular reference to laser power, pulse energy, and overlapping factor, can reduce the HAZ extension: moreover, the use of cryogenic parameters seems to be a promising process development.

### 4.2. Electrical Discharge Machining

Electrical discharge machining (EDM) can contribute to avoid the typical problems of conventional FRP machining. The EDM process does not involve mechanical energy, thus hardness, strength, toughness, or abrasiveness of the work material does not affect the machining process. Accordingly, the EDM capability to remove material from electrically conducting materials without the application of mechanical force can overcome many of the difficulties that are seen in conventional machining of CFRP composites such as drilling. Although EDM is a widely used process in the die and tool industry, its use in machining CFRP is very limited. Although some studies have addressed the machinability of CFRP with EDM, the material removal mechanisms and the machining damage that are associated with them are not well understood. The studies which addressed the machinability of CFRP by EDM confirmed the feasibility of the process, identified process parameters important for achieving machinability, and pointed out the potential problems arising from its application. Research studies on the proper process parameters for EDM of FRP composites revealed that there is a very specific range of parameters at which the workpiece can be machined. Typically, for these materials, the matrix material is non-conductive; however, if the fibre material is conductive, as in the case of CFRP, then the carbon fibres allow for generating the sparks that are required for material removal by EDM. Guu et al. [70] performed drilling of carbon composites and evaluated delamination damage and surface roughness. It was found that delamination has its highest levels at the highest pulse current. The studies in [70,71] addressed the issue of electrode wear and the effects of current and polarity, and concluded that copper electrodes provide less wear than graphite, while graphite electrodes with positive polarity provide the highest material removal rates. The studies in [70] reported that material removal takes place by melting and vaporization and that discharge current is the most significant parameter affecting machinability. It has been demonstrated that, with increasing pulse energies, both a high tool wear and low surface finish can be produced [72].

Habib and Okada [73] studied the feasibility of CFRP machining via EDM process under various cutting conditions such as peak current, pulse-on time, pulse-off time, open-circuit voltage, and electrode rotation. The experimental results confirmed that the material removal rate increases with pulse-on time, pulse-off time, peak current, speed of electrode rotation, and open circuit voltage until it reaches a maximum value. Graphite electrodes provided higher material removal rates than copper electrodes, but the latter produced a smoother surface roughness. The surface roughness increased with pulse-on time, peak current, and open circuit voltage, and decreased with pulse-off time and electrode rotation speed until reaching a minimum value. Therefore, it has been shown that CFRP composites can only be adequately machined within limited EDM parameter ranges.

### 4.3. Other Unconventional Machining Processes

To reduce the problems related to conventional machining of FRP composites, other unconventional processes were investigated in the literature, including abrasive machining methods such as abrasive water jet machining or vibration assisted cutting and hybrid machining methods such as rotary ultrasonic machining.

Feng et al. [74] experimentally investigated the feasibility of CFRP machining by using rotary ultrasonic machining (RUM). Chips, edge chipping, surface roughness, tool wear, and thrust force were measured. The effects of RUM process variables (rotation speed, vibration amplitude, and feedrate) on thrust force and surface roughness were studied, and the results showed that RUM could be used to drill holes in CFRP with high productivity and low tool wear, producing a better surface with high rotation speed and low feed rate.

Hybrid machining methods merging diverse technologies have been proposed. In [75], vibration-assisted drilling (VAD) was studied to reduce thermal and mechanical defects in drilling of FRP composites. The VAD intermittent cutting redistributed the cutting energy over the engagement cycles, thus enhancing the tool cooling (cutting temperature −50%) and reducing the axial force component (−40%).

Xu et al. [76] studied the elliptic vibration-assisted (EVA) cutting of FRP composites to investigate the chip formation mechanism and its influence on cutting forces. The key factors governing the cutting forces, such as the depth of cut, feed rate, tool vibration frequency, and amplitude were taken into account, and the study revealed that fibre orientation significantly affects the chip formation and cutting forces. When a fibre orientation is less than 90°, chipping mainly occurs through bending-induced fracture of fibres; when it is beyond 90°, chipping mostly occurs by crushing the fracture of fibres. When compared with a traditional cutting process, the EVA cutting can minimize the fibre orientation effect through localized fibre fracture.

Water jet machining of FRP composites was investigated by Voit et al. [77]. A methodological approach for the study of process parameters in water jet machining of unidirectional carbon fibre fabrics was presented. Improved parameters—nozzle distance, forward speed, and water pressure—were determined for unidirectional carbon fibre textiles.

Wong et al. [78] studied the trimming of hybrid carbon/glass FRP composites through abrasive water-jet machining (AWJM) to examine the main geometrical defects that can be produced by this process, i.e., kerf taper and delamination. Stand-off distance was identified as the dominating factor, followed by traverse rate, in order to minimize the kerf ratio. The delamination damage of the hybrid composites was more severe on the entrance side as compared to the bottom side. The abrasive flow rate was the predominant factor for delamination damage followed by traverse rate and hybrid pressure. Minimum delamination damage can be achieved by increasing the kinetic energy of abrasive water-jet stream when impacting the composite under a lower speed.

## 5. Conclusions

The phenomena responsible for material removal in cutting FRP composite materials are fundamentally different from those of conventional metals and their alloys. The FRP material behaviour is non-homogeneous and depends on diverse reinforcement and matrix properties, reinforcement architecture and orientation, and the relative content of matrix and reinforcement.

Besides tool geometry and process parameters, the chip formation mechanisms for FRP composites are critically governed by the fibre orientation with respect to the cutting direction: disadvantageous fibre orientations can result in severe damage to the workpiece.

The machining of FRP composite materials imposes special demands on the cutting tool geometry and material: new tool materials (PCD, CBN, DLC, TiN, and other coatings), advanced tool design, and optimal cutting parameters selection are required to substantially improve tool life which can be rather short due to the highly abrasive nature of the reinforcement materials.

In the present review, the key issues concerning the machining of fibre reinforced plastic composite materials were discussed with reference to the main recent research works in the field, when considering both conventional and unconventional machining processes and reporting the more recent research achievements. Conventional cutting processes, such as milling, turning, drilling, and trimming have been investigated with the aim to reduce the damage that is induced in the workpiece material, such as delamination, uncut fibres, fuzzying, cracking, as well as to improve the surface roughness of the machined surface. Deep studies on chip formation and machining process optimisation are still required in order to improve the quality of machined parts.

As regards the main process developments for the different machining processes, the main results characterizing the recent research works were presented.

The research studies that were carried out on turning of FRP composites highlighted the need to select low feed values to optimize machined surface roughness and showed the increasing use of PCD cutting tools, characterized by high strength, toughness, hardness, and thermal shock resistance.

As regards the milling process, different process developments were proposed in the recent literature to reduce the typical damages, such as delamination and fibre protrusions at different ranges of fibre orientation angle. Some authors suggested to perform an initial scoring along the contour via grinding or laser cutting. Others proposed oscillated milling to reduce delamination, as the very recent research studies reported a significant improvement of the milled surface quality.

Moreover, the investigation of high speed milling is a promising field of research, as the recent experimental results showed that higher cutting speeds lead to decreased process forces, allowing for prolonging tool life or increasing the feed rate. Another interesting opportunity is the employment of minimum quantity lubrication, which may allow for prolonging tool life and reducing the environmental impact, related to a more intensive use of cutting fluids.

Drilling is probably the machining process that received most attention by the researchers, due to its wide employment industry for the realization of mechanical joints. Several research studies were focused on the selection of the proper process parameters, as well as tool geometry and material with the aim to reduce critical damages, such as peel-up and push-out delamination, and others focused on the suitable methodologies to measure these defects and to properly characterize the hole quality. High-speed drilling has been investigated, revealing that the delamination tendency decreases with an increase in cutting speed. A recent development that is claiming increasing attention in drilling of FRP composites is the orbital drilling process, requiring however longer processing times. As an interesting alternative to conventional drilling tools, the use of diamond grinding tools for drilling of FRP composites is also under investigation.

As an alternative to conventional cutting processes, in the last years, several research efforts have been also spent to investigate the applicability of unconventional machining technologies, such laser beam processes, electrical discharge machining, or abrasive waterjet machining, with the aim to avoid the typical problems related to high tool wear or material damage due to mechanical forces exerted in traditional cutting.

As regards laser beam machining of FRP composites, an accurate selection of the process parameters, with particular reference to laser power, pulse energy, and overlapping factor, can reduce the extension of the heat affected zone: moreover, the use of cryogenic parameters seems to be a promising process strategy. As regards the machinability of CFRP by EDM, the research studies confirmed the feasibility of the process, and revealed that there is a very specific range of parameters at which the workpiece can be machined obtaining acceptable surface finish.

Finally, the research works that are presented in this review highlighted that hybrid machining methods merging diverse technologies have been recently proposed: an example is vibration-assisted drilling, which was studied to reduce thermal and mechanical defects in the drilling of FRP composites.
